# Short-Term Effects of Elexacaftor/Tezacaftor/Ivacaftor Combination on Glucose Tolerance in Young People With Cystic Fibrosis—An Observational Pilot Study

**DOI:** 10.3389/fped.2022.852551

**Published:** 2022-04-21

**Authors:** Insa Korten, Elisabeth Kieninger, Linn Krueger, Marina Bullo, Christa E. Flück, Philipp Latzin, Carmen Casaulta, Claudia Boettcher

**Affiliations:** ^1^Division of Paediatric Respiratory Medicine and Allergology, Department of Paediatrics, Inselspital, Bern University Hospital, University of Bern, Bern, Switzerland; ^2^Department of Paediatric Endocrinology, Diabetology and Metabolism, Department of Paediatrics, Inselspital, Bern University Hospital, University of Bern, Bern, Switzerland; ^3^Department of BioMedical Research, Bern University Hospital, University of Bern, Bern, Switzerland

**Keywords:** cystic fibrosis, elexacaftor/tezacaftor/ivacaftor therapy, oral glucose tolerance test (OGTT), continuous glucose monitoring, glucose tolerance

## Abstract

**Background:**

The effect of elexacaftor/tezacaftor/ivacaftor (ELX/TEZ/IVA) on glucose tolerance and/or cystic-fibrosis-related diabetes (CFRD) is not well understood. We performed an observational study on the short-term effects of ELX/TEZ/IVA on glucose tolerance.

**Methods:**

Sixteen adolescents with CF performed oral glucose tolerance tests (OGTT) before and 4–6 weeks after initiating ELX/TEZ/IVA therapy. A continuous glucose monitoring (CGM) system was used 3 days before until 7 days after starting ELX/TEZ/IVA treatment.

**Results:**

OGTT categories improved after initiating ELX/TEZ/IVA therapy (*p* = 0.02). Glucose levels of OGTT improved at 60, 90, and 120 min (*p* < 0.05), whereas fasting glucose and CGM measures did not change.

**Conclusion:**

Shortly after initiating ELX/TEZ/IVA therapy, glucose tolerance measured by OGTT improved in people with CF. This pilot study indicates that ELX/TEZ/IVA treatment has beneficial effects on the endocrine pancreatic function and might prevent or at least postpone future CFRD.

## Introduction

Within the last years, CFTR modulators that target the cystic fibrosis transmembrane conductance regulator (CFTR) ion channel have become available to many people with cystic fibrosis (CF) (1). They may either potentiate CFTR channel activity at the epithelial cell surface or correct the defect by helping CFTR reach the cell surface (2–4). The latest and most efficient CFTR drug combines elexacaftor, tezacaftor, and ivacaftor (ELX/TEZ/IVA, one potentiator and two correctors, respectively). Several randomized controlled trials showed excellent results concerning the safety and efficacy of ELX/TEZ/IVA (5–7) and improved respiratory scores such as lung function (1, 8).

However, little is known about the effects of ELX/TEZ/IVA on other organs affected by CFTR dysfunction. Cystic fibrosis-related diabetes (CFRD) represents one of CF’s most frequent extrapulmonary complications and is associated with lung function decline, poor nutritional status, and increased mortality (9–11). Reductions in islet size and beta-cell area seem to lead to glucose responsiveness abnormalities and insulin secretory defects (12, 13). However, the exact mechanisms leading to islet dysfunction are still unclear. Whilst several studies have shown CFTR channel activity in rodent and human islet cells (13, 14), others reported minimal or absent CFTR expression in human pancreatic endocrine cells, suggesting that insulin secretory defects are due to islet mass reduction, intra-islet inflammation, or inflammatory mediators (12, 15, 16).

Favoring the hypothesis of CFTR expression in islet cells, it is speculated that CFTR modulators may positively affect glucose tolerance and insulin secretion. Studies investigating mono- or dual-CFTR modulators present contradictory results. While several authors report improved glucose tolerance after starting therapy with ivacaftor (17–22) or lumacaftor/ivacaftor (23), others did not find an impact on glucose or insulin levels regarding CFTR modulators (24–26). A study in adults with CF—with and without a diagnosis of CFRD—reported an improvement of hyperglycemia and glycemic variability after initiation of ELX/TEZ/IVA (27).

We hypothesize a beneficial effect of ELX/TEZ/IVA treatment on glucose tolerance and insulin secretion: the aim of our study was thus to investigate the short-term effects of ELX/TEZ/IVA on glucose tolerance in insulin-naïve adolescents with CF by assessing oral glucose tolerance test (OGTT) and continuous glucose monitoring (CGM) before and after starting ELX/TEZ/IVA therapy.

## Materials and Methods

### Study Population

In Switzerland, ELX/TEZ/IVA was approved by the Swissmedic in December 2020 for all CF individuals 12 years or older with at least one F508del CFTR mutation. All patients in the CF outpatient clinic of the University Children’s Hospital of Bern, Switzerland, qualifying for therapy with ELX/TEZ/IVA, were notified about approval of ELX/TEZ/IVA during routine outpatient visits in the first quarter of 2021. We recommended ELX/TEZ/IVA to all eligible patients and asked patients, parents, or caregivers to participate in our study. We excluded patients with known CFRD to include insulin naïve participants only.

The study was performed in the University Children’s Hospital of Bern, University of Bern, Switzerland, and approved by the Ethics committee of Bern, Switzerland (ID 2021-00982). Informed written consent was obtained from all parents or caregivers and teenagers ≥ 14 years.

### Study Design

**FIGURE 1:**
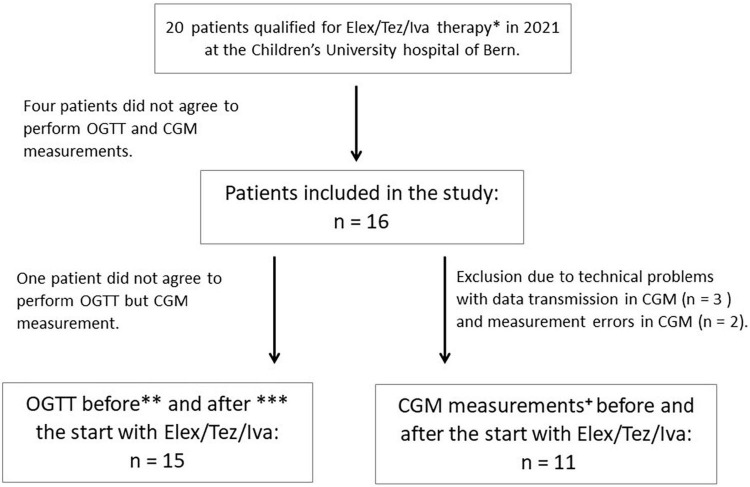
Flowchart of study design. *Patients with known CFRD were excluded. **OGTT was performed 3 days to 6 weeks before the start with ELX/TEZ/IVA, except for three patients in whom OGTT was performed earlier (10 weeks, 4 months, and 6 months before the start) due to organizational reasons during routine clinical visits and disagreement of the patients to perform another OGTT. ***OGTT was performed 3–6 weeks after starting ELX/TEZ/IVA treatment, except for two patients in whom OGTT was performed after 8 and 10 weeks, respectively. All patients continued using ELX/TEZ/IVA until the second OGTT was performed. ^+^CGM measures were performed 3 days before and 7 days after starting with ELX/TEZ/IVA. In one patient, CGM was only installed 2 days before the initiation of ELX/TEZ/IVA therapy due to organizational reasons in routine clinical visits.

An OGTT was performed in median (IQR) 3 days (3–42) before (first study visit) and 26 (24–40) days after (second visit) initiating ELX/TEZ/IVA treatment. CGM measures were recorded 3 days before until 7 days after the start with ELX/TEZ/IVA therapy. Height and weight were obtained on both study visits, lung function measurements, HbA1c, and a sweat chloride test was performed (Figure 1).

### Oral Glucose Tolerance Tests Measures

A standardized 3-h OGTT was performed before and after initiating ELX/TEZ/IVA therapy. After an overnight fast, an intravenous line was placed for blood draws. Participants consumed 1.75 g/kg (maximum 75 g) dextrose. Blood sampling was performed at baseline and 60, 90, 120, and 180 min (min) post-dextrose ingestion. We determined plasma glucose concentration, serum insulin levels, and c-peptide levels (28), as well as HbA1c. OGTT results were categorized into normal glucose tolerance (NGT) [normal fasting (<5.6 mmol/L) and 2-h (<7.8 mmol/L) plasma glucose], indeterminate glucose tolerance (INDET) (normal fasting and 2-h plasma glucose, 1-h plasma glucose value of ≥ 11.1 mmol/L), impaired fasting glucose (IFG) (fasting glucose 5.6–6.9 mmol/L, normal 2-h plasma glucose), impaired glucose tolerance (IGT) (fasting glucose 5.6–6.9 mmol/L, 2-h plasma glucose 7.8–11.0 mmol/L), and CFRD (fasting glucose > 6.9 mmol/L, 2-h plasma glucose ≥ 11.1 mmol/L). The classification was based on the American Diabetes Association (ADA) criteria (29) and international guidelines for screening and treating CFRD (30). Due to low numbers of IFG, IGT and IFG were both categorized into the IGT group.

### Continuous Glucose Monitoring Measures

CGM systems track glucose levels in interstitial fluid via a sensor inserted into the subcutaneous fat tissue. A transmitter connected to the sensor wire sends real-time data wirelessly to a receiver. The receiver displays real-time glucose levels and historical trends and provides acoustic alarms when certain glucose thresholds (e.g., hypo- and hyperglycemia) are reached.

Participants wore a Dexcom G6 CGM-System (Dexcom, Inc., San Diego, CA, United States) at the upper arm inserted by specialized diabetes nurses using an automatic applicator. Families were instructed on how to use the CGM device and react to alarms personally. Additional written information was provided. As the exact time point of medication intake on Day 1 was not assessed, we excluded CGM measures of this day to include only measurements prior to or after the start of therapy. Furthermore, we included only days of measurement where all individuals wore a sensor to have an equal number of days for evaluation. Thus, for the standardized evaluation of glucose data by CGM, we included the following measures: (a) glucose measurements of Day 1 (start 4 pm) to Day 3 (stop 12 pm) before initiating ELX/TEZ/IVA therapy, approximately 2.5 days of measuring time; (b) glucose measurements of Day 2 post-ELX/TEZ/IVA (start 12 pm) and Day 6 (stop 12 pm) for analysis after the initiation of ELX/TEZ/IVA therapy, resulting in a total of 5 days of measuring time. We used all available measuring points from CGM measurements generating mean, minimum, and maximum glucose values. Furthermore, we calculated the percentage of time of glucose levels within the following categories: very low (≤2.7 mmol/L), low (≥2.8 and ≤3.3 mmol/L), normal (≥3.4 and ≤7.7 mmol/L), high (≥7.8 and ≤11.1 mmol/L), and very high (≥11.2 mmol/L), according to guidelines (31, 32) and as implemented in previous studies (24, 27).

### Lung Function Testing

Ventilation inhomogeneity was derived from N2-multiple breath washout (MBW) measurements performed according to guidelines using the manufacturer’s software (Spiroware V 3.2.1, Eco Medics AG, Duernten, Switzerland; reloaded with Spiroware V 3.3.1) (33, 34) with the primary outcome lung clearance index (LCI). N_2_MBW trials were quality controlled, and mean values from tests with at least two trials of acceptable quality were included for analysis (35). Spirometry and body plethysmography (Jaeger MasterScreen Body plethysmography, CareFusion, Hochberg, Germany) was performed according to ERS/ATS guidelines (36). Outcomes were forced expiratory volume at 1 s (FEV_1_), the ratio of FEV_1_/FVC (forced vital capacity), and mid forced expiratory flow (MFEF). Z-scores for spirometry were derived from published reference equations; normal lung function’s upper and lower limits were defined as ±1.64 z-scores (37). Outcome parameters from body plethysmography were residual volume (RV)/total lung capacity (TLC) and specific resistance (sReff).

### Statistical Analysis and Power Calculation

Descriptive statistics were expressed as median and interquartile ranges or numbers and percentages, as appropriate. We used the Wilcoxon’s signed rank test to compare values before and after ELX/TEZ/IVA initiation. To investigate associations between the different parameters, we used Spearman rank correlation, Kruskal–Wallis test, and Fisher’s exact test. The area under the curve (AUC) for glucose, insulin, and plasma C-peptide levels was calculated using the trapezoidal estimation. A *p*-value of ≤ 0.05 was considered statistically significant.

We used existing data of the effect of mono- or dual-CFTR modulators on glucose tolerance and insulin secretion for sample size calculation (17, 23). Assuming an alpha level of 0.05 (one-sided), a statistical power of 80%, an effect size (= difference of 2-h glucose values before/after starting ELX/TEZ/IVA treatment) of 2.0 mmol/L, and a standard deviation (SD) of 2.8 mmol/L, we estimated a minimal sample size of *n* = 14 (two-sample paired-means *t*-test). Statistical analyses were performed using Stata™ (Stata Statistical Software: Release 13; StataCorp LP, College Station, TX, United States). Figures were generated using GraphPad Prism 8.0 (GraphPad Software, San Diego, CA, United States).

## Results

### Study Population

In total, 16 adolescents with CF with a median age (IQR) of 13.8 years (13.0; 15.4) were included in the study (Figure 1). Baseline characteristics were assessed at the first study visit. All participants performed lung function measurements before and 4–6 weeks after initiating ELX/TEZ/IVA treatment. Details on demographic data are given in Table 1.

**TABLE 1:** Demographic data of study population.

		Before ELX/TEZ/IVA therapy	Under ELX/TEZ/IVA therapy	*P*-value
**Demographics** ***n* = 16**	Sex (m)	6 (37.5)		
	Age (years)	13.8 (13.0; 15.4)		
	Weight (z-score)	−0.29 (−1.08; 0.23)	−0.25 (−0.94; 0.28)	0.5
	BMI (z-score)	−0.47 (−0.74; 0.33)	−0.25 (−0.83; 0.38)	0.09
	Pancreatic insufficiency	16 (100)		
**CFTR variant**	F508 homozygot	9 (56)		
	F508 heterozygot	7 (44)		
	Previous modulator use	5 (31)		
	− Ivacaftor/Tezacaftor	4 (25)		
	− Ivacaftor/Lumacaftor	1 (6)		

*Results are displayed as median (IQR) or total numbers (%) as appropriate. CFTR, cystic fibrosis transmembrane conductance regulator.*

### Glucose Tolerance Before and After the Start With Elexacaftor/Tezacaftor/Ivacaftor

**FIGURE 2:**
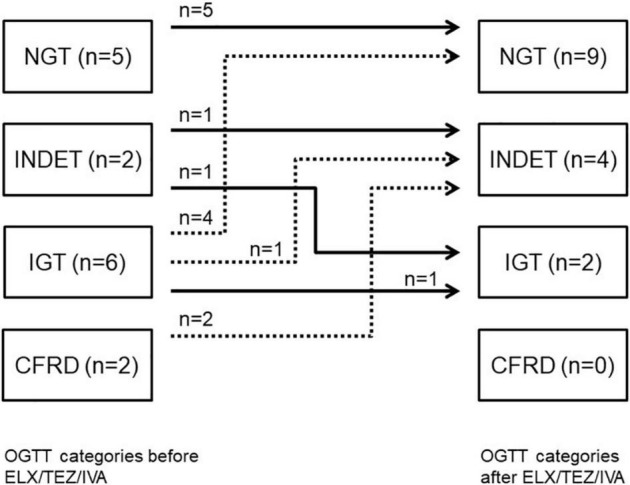
OGTT categories before and after starting ELX/TEZ/IVA treatment in people with cystic fibrosis. Numbers of study participants and OGTT categories and individual changes after starting ELX/TEZ/IVA treatment. OGTT, oral glucose tolerance test; NGT, normal glucose tolerance; INDET, indeterminate glucose tolerance; IFG, impaired fasting glucose; IGT, impaired glucose tolerance; CFRD, CF-related diabetes.

OGTT was performed in 15 people with CF in median (IQR) 3 (2–42) days (IQR) before and 26 (24–40) days after starting treatment with ELX/TEZ/IVA. OGTT improved after starting with ELX/TEZ/IVA (*p* = 0.02). Before ELX/TEZ/IVA treatment, two participants were diagnosed with (previously unknown) CFRD, and six could be categorized as IGT, two as INDET, and five as NGT. After treatment, no participant fell into the category CFRD, two into the category IGT, four into INDET, and nine into NGT (Figure 2). Thus, seven participants improved in OGTT, and seven participants remained stable. Of the latter, five participants had a normal result (NGT) in both tests. One participant changed from INDET to IGT (Figure 2).

**FIGURE 3:**
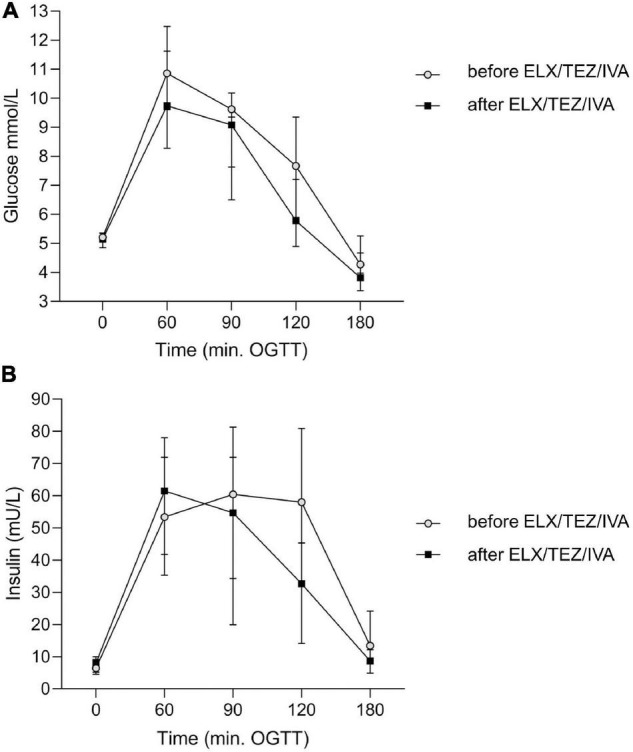
Time course of plasma glucose **(A)** and insulin **(B)** levels before and after the start of ELX/TEZ/IVA treatment at different time points of the OGTT in people with cystic fibrosis. After starting ELX/TEZ/IVA treatment **(A)**, plasma glucose levels (in mmol/L) were lower at 60, 90, and 120 min of OGTT (all *p* < 0.05), but no difference was found for fasting glucose and glucose levels at 180 min of OGTT. **(B)** Plasma insulin levels (in mU/L) were lower at 120 min (*p* = 0.04) and 180 min (*p* = 0.006), whereas no differences were found for fasting and early insulin secretion. OGTT, oral glucose tolerance test.

**FIGURE 4:**
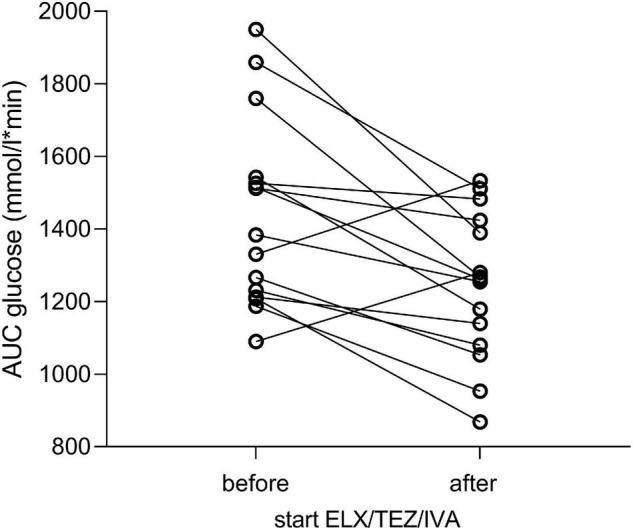
Area under the curve of plasma glucose levels before and after initiating ELX/TEZ/IVA therapy in people with cystic fibrosis. The area under the curve (AUC) decreased for plasma glucose (in mmol/l*min) after introducing ELX/TEZ/IVA (*p* = 0.008).

We compared glucose, insulin, and plasma C-peptide levels at time points 0, 60, 120, and 180 min of OGTT, respectively (Table 2 and Figure 3). After starting with ELX/TEZ/IVA, compared to pre-treatment measurements, plasma glucose levels were lower at 60, 90, and 120 min of OGTT, but no difference was found for fasting glucose and glucose levels at 180 min of OGTT (Figure 3A). Insulin and C-peptide levels were lower at 120 min and 180 min, whereas no differences were found for fasting and early secretion of insulin (at 60, 90 min) and C-peptide (at 60, 90 and 120 min) (Figure 3B). The AUC decreased for plasma glucose (Figure 4) and insulin after introducing ELX/TEZ/IVA (Table 2). Results did not change when excluding participants with NGT (*n* = 5) before initiating ELX/TEZ/IVA therapy (difference in OGTT categories: *p* = 0.02). HbA1c values did not change before and after starting with ELX/TEZ/IVA (Table 2).

**TABLE 2:** Differences in glucose tolerance (OGTT and CGM) and lung function before and after initiation of ELX/TEZ/IVA therapy in people with cystic fibrosis.

		Before ELX/TEZ/IVA therapy	Under ELX/TEZ/IVA therapy	*P*-value
**OGTT categories**	NGT	5 (33.3)	9 (60)	**0.02**
**and measures**	INDET	2 (13.3)	4 (26.6)	
***n* = 15**	IGT/IFG	6 (40)	2 (13.3)	
	CFRD	2 (13.3)	0	
	Fasting plasma glucose (mmol/L)	5.21 (5.12; 5.35)	5.15 (4.85; 5.35)	0.2
	60 min plasma glucose (mmol/L)	10.86 (9.61; 12.48)	9.74 (8.28; 11.62)	**0.03**
	90 min plasma glucose (mmol/L)	9.62 (7.63; 11.38)	9.08 (6.5; 10.18)	**0.04**
	120 min plasma glucose (mmol/L)	7.67 (5.9; 9.35)	5.78 (4.9; 7.2)	**0.03**
	180 min OGTT plasma glucose (mmol/L)	4.27 (3.98; 5.25)	3.82 (3.37; 4.67)	0.6
	Average AUC blood glucose (mmol/L*min)	1383.75 (1212.45; 1542.45)	1262.10 (1079.85; 1423.5)	**0.008**
	Fasting insulin (mU/L)	6.4 (5.2; 10)	8 (4.5; 9.1)	0.7
	60 min insulin (mU/L)	53.4 (41.8; 71.9)	61.5 (35.3; 78)	0.6
	90 min insulin (mU/L)	60.4 (19.9; 80.9)	54.7 (34.3; 81.3)	0.7
	120 min insulin (mU/L)	58 (33.2; 79.5)	32.65 (14.1; 45.3)	**0.01**
	180 min insulin (mU/L)	13.4 (8.6; 24.1)	8.7 (4.9; 12.1)	**0.006**
	Average AUC blood insulin (mU/L*min)	8014.5 (5,160; 10,434)	6,924 (4,353; 9,330)	**0.02**
	Fasting plasma C-peptide (ng/ml)	1.16 (0.98; 1.53)	1.26 (0.93; 1.62)	0.6
	60 min plasma C-peptide (ng/ml)	8.46 (6.2; 9.7)	6.92 (4.68; 7.7)	0.8
	90 min plasma C-peptide (ng/ml)	8.62 (5.41; 9.81)	7.85 (5.16; 10.8)	0.5
	120 min plasma C-peptide (ng/ml)	8.12 (5.54; 9.8)	5.95 (3.57; 7.85)	0.08
	180 min plasma C-peptide (ng/ml)	3.52 (2.09; 4.97)	2.02 (1.58; 3.18)	**0.005**
	Average AUC plasma C-peptid e (ng/ml*min)	1067.4 (900.9; 1196.7)	930.45 (736.05; 1145.85)	0.05
**CGM measures**	Average sensor glucose (mmol/L)	6.68 (6.22; 6.94)	6.6 (6.06; 7.46)	0.6
***n* = 11**	Minimum sensor reading (mmol/L)	4 (3.1; 4.2)	4 (3.7; 4.5)	0.2
	Maximum sensor reading (mmol/L)	13.15 (12.2; 14.0)	13.1 (12.4; 14.9)	0.3
	% time glucose ≥ 11.2 (very high) (mmol/L)	3.0 (0.6; 4.46)	1.15 (0.84; 2.08)	0.3
	% time glucose ≥ 7.7 and ≤ 11.1 (high) (mmol/L)	17.56 (9.78; 21.73)	18.0 (12.28; 27.09)	0.2
	% time glucose ≥ 3.4 and ≤ 7.7 (normal) (mmol/L)	81.11 (77.38; 85.97)	81.13 (68.83; 86.6)	0.5
	% time glucose ≥ 2.8 and ≤ 3.3 (low) (mmol/L)	0 (0; 0.15)	0 (0; 0)	0.4
	% time glucose ≤ 2.7 (very low) (mmol/L)	0 (0; 0)	0 (0; 0)	−
**Additional laboratory**	HbA1c%	5.7 (5.4; 5.9)	5.6 (5.5; 5.9)	0.6
**results** ***n* = 16**	HbA1c ≥ 5.8% (*n*,%)	7 (47)	6 (40)	0.6
	Sweat chloride (mmol/l)	95 (93; 104)	51 (32; 59)	**0.002**
**Lung function**	LCI (TO)	8.05 (6.82; 10.22)	6.84 (6.39; 7.89)	**0.003**
**data**	FEV_1_ (z-score)	−0.71 (−2.39; −0.32)	−0.39 (−1.08; 0.4)	**0.007**
***n* = 16**	MFEF (z-score)	−0.59 (−2.05; 0.15)	0.07 (−0.46; 0.9)	**0.001**
	FEV_1_/FVC (z-score)	−0.42 (−1.23; −0.02)	0.31 (−0.33; 0.78)	**0.0009**
	RV/TLC (%predicted%)	27.76 (21.12; 37.04)	23.09 (19.5; 27.63)	**0.006**
	sReff (%predicted)	179 (125.5; 249.5)	144 (102; 175)	**0.01**

*Differences between OGTT and CGM measures as well as additional laboratory results and lung function data in people with cystic fibrosis (CF) before and after the initiation of elexacaftor/tezacaftor/ivacaftor (ELX/TEZ/IVA) treatment. Results are displayed as median (IQR) or total numbers (%) as appropriate. CFTR, cystic fibrosis transmembrane conductance regulator; CGM, continuous glucose monitoring; OGTT, oral glucose tolerance test; NGT, normal glucose tolerance; INDET, indeterminate glucose tolerance; IFG, impaired fasting glucose; IGT, impaired glucose tolerance; CFRD, CF-related diabetes; AUC, area under the curve; LCI, lung clearance index; FEV_1_, forced expiratory volume at 1 s; FVC, forced vital capacity; MFEF, mid expiratory flow; RV/TLC, ratio of residual volume over total lung capacity; staf, specific resistance. Significant p-values (≤0.05) are marked in bold.*

### Continuous Glucose Monitoring

**FIGURE 5:**
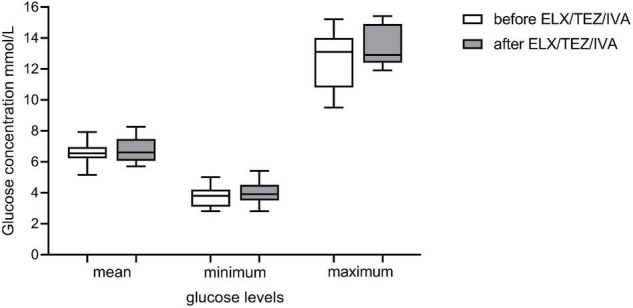
Glucose levels of continuous glucose monitoring (CGM) prior and after initiation of ELX/TEZ/IVA therapy in people with cystic fibrosis. Mean, minimum and maximum glucose levels (mmol/l) of CGM measurements prior (measurements of 2 days) and after (measurements of 5 days) starting ELX/TEZ/IVA therapy. Displayed are boxplots with medium and interquartile range.

CGM was performed in 11 participants 3 days (median, min–max 2–4) before and 7 (6–7) days after the initiation of ELX/TEZ/IVA therapy (Table 2). We excluded the measurements on the first treatment day to standardize analyses, as the precise time of first medication use was not documented. To have genuinely only readings before and after initiation, we analyzed measurements from 2 days before and 5 days after therapy started (for details, see “Materials and Methods” section). We calculated mean, minimum, and maximum glucose levels before and after starting with ELX/TEZ/IVA and found no difference (Figure 5). Average sensor readings were within normal glucose ranges before and after the start with ELX/TEZ/IVA [median (IQR) 6.68 (6.22; 6.94) mmol/L and 6.60 (6.06; 7.46) mmol/L, *p* = 0.60, respectively]. As described in detail in the methods section, we classified glucose levels into different categories (very low, low, normal, high, and very high). We did not find a difference in the percentage of the respective glucose level time before and after ELX/TEZ/IVA initiation. No episodes of hypoglycemia (very low and low category) were observed after starting ELX/TEZ/IVA treatment. Episodes of hyperglycemia were similar before and after starting ELX/TEZ/IVA treatment (Table 2).

### Lung Function and Additional Analyses

Lung function measurements and additional analyses were performed on the day of OGTT. All lung function parameters from N_2_-MBW (LCI), spirometry (FEV_1_, FEV_1_/FVC, and MFEF), and body plethysmography (RV/TLC, sReff) improved under ELX/TEZ/IVA treatment (Table 2). Sweat chloride was pathologic (>60 mmol/L) in all people with CF before ELX/TEZ/IVA therapy and improved in all, normalizing (<30 mmol/L) in three and reaching borderline results (30–60 mmol/L) in ten. Weight and BMI did not change before and under ELX/TEZ/IVA (Table 2). Changes in OGTT [categorized into (i) improvement, (ii) no change and (iii) deterioration], plasma glucose, insulin, and C-peptide levels did not correlate with lung function parameters (meaning differences between the first and second measurement) and were not associated with the reduction of sweat chloride (data not shown).

## Discussion

### Summary

In this observational pilot study, glucose tolerance measured via OGTT improved in adolescents with CF shortly after initiating ELX/TEZ/IVA treatment, whereas glycemic profiles assessed by CGM did not show any changes. Lung function parameters and sweat chloride levels improved as well. Data of this observational pilot study indicate that ELX/TEZ/IVA improves respiratory outcomes—as shown previously (1, 38)—in people with CF and positively affects endocrine pancreatic function.

### Comparison With Literature

In our study, OGTT results generally improved, as did plasma glucose levels at 60, 90, and 120 min, and late plasma insulin levels (90 and 120 min) under ELX/TEZ/IVA therapy. Our data is in line with a previous study investigating glucose tolerance before and after 1 year of lumacaftor/ivacaftor therapy in 40 adult people with CF with IGT or newly diagnosed and untreated CFRD (23). The authors reported an improvement of OGTT overall and in 2 h plasma glucose. Improvement of glycemic control or insulin secretion was observed under ivacaftor therapy (22, 39). In our study population, there was no new diagnosis of CFRD under ELX/TEZ/IVA treatment, but we could record a resolution of CFRD into IGT. These results align with data from patients with ivacaftor therapy, in whom one-third of patients with CFRD showed resolution (40). We found no difference in fasting glucose and early insulin secretion or improved HbA1c values after ELX/TEZ/IVA treatment initiation. Others found improvement under ivacaftor in all parameters mentioned above but not in OGTT values in teenage and adolescent patients with CF (18, 24). Further, a small study in five patients with NGT, IGT, or CFRD reported improved insulin response in OGTT 4 weeks after starting with ivacaftor, whereas glucose levels did not improve (17). However, several studies investigating glucose tolerance under lumacaftor/ivacaftor treatment (from 4 weeks up to 12 months) could not find differences for any assessed parameters: OGTT, plasma glucose, insulin, and C-peptide levels (25, 26, 41). We did not observe differences in CGM before and after the start with ELX/TEZ/IVA, our results are in line with a study investigating CGM before and after the start with ivacaftor in nine teenagers (24). A recent study in adults using ELX/TEZ/IVA reported improvement of CGM data after initiation of therapy. However, the main improvement could be found in patients with CFRD. Furthermore, CGM measures in this study were performed 3–12 months after the start with ELX/TEZ/IVA; thus, a further improvement in our cohort is possible (27).

Several studies investigating people with CF with impaired glucose tolerance reported low early-phase and high late-phase insulin secretion (42, 43). Our study showed that the late-phase insulin levels decreased after ELX/TEZ/IVA therapy initiation—an effect we interpret as insulin-resistance-improvement: less insulin is needed for lower glucose levels, and the initial insulin secretion is sufficient. The observed insulin secretion pattern after starting ELX/TEZ/IVA therapy approximates the pattern described in healthy people (43). The lower insulin levels under ELX/TEZ/IVA combined with lower glucose concentrations could be interpreted as higher insulin sensitivity.

The study population had no higher incidence of hypo- or hyperglycemic episodes under ELX/TEZ/IVA treatment.

The discrepancy between studies might be due to differences in study design, small participant numbers, and different CFTR modulators/correctors or combinations studied. Studies on lumacaftor/ivacaftor in patients with F508del mutation showed fewer CFTR activating effects than ivacaftor alone in patients with G551D mutation (44), which might explain the missing effect on glucose tolerance in studies investigating lumacaftor/ivacaftor.

All lung function parameters improved after initiation of ELX/TEZ/IVA. Our results are comparable with those of a larger previous study in children (45). Median improvement of lung function data (e.g., LCI and FEV1) in our study is in line with mean improvement in this larger cohort. This is also true for the improvement of sweat chloride (45).

### Physiological Considerations and Clinical Implications

The mechanisms of how CFTR modulators may affect the pancreatic function and thus glucose homeostasis are not fully understood. Insulin deficiency is the primary defect in CFRD, but insulin resistance and impairment of the entero-insular axis play contributory roles (46). A dysbalance between pancreatic β-cell mass and insulin secretion (42, 47), and pancreatic islet loss are discussed as underlying pathophysiology (12). CFTR protein expression in pancreatic α- and β-cells and an early presence of abnormalities in insulin secretion imply a direct role of CFTR dysfunction (13, 14, 48). CFTR seems to play a role in glucose-induced electrical activities leading to CFTR-caused defect in β-cells and diminished insulin secretion, which was shown to be restored by lumacaftor *in vitro* (48). However, others did not detect CFTR activity in pancreatic cells (12), postulating that CFTR function indirectly impacts insulin secretion: an improved incretin secretion by gastrointestinal neuroendocrine cells and decreased inflammation could, e.g., lead to indirect β-cell effects (16). Another possible mechanism could be a relief of islet inflammation by CFTR restoration, as CF islets contain inflammatory interleukins (16, 49). CFTR restoration might improve the local environment of islets by enhancing pancreatic ductal function (16). Furthermore, one mechanism that leads to insulin resistance is an impaired insulin-induced glucose transporter 4 (GLUT), inhibiting glucose entering into dependent cells and impairing subsequent signaling pathways (50). Recently, Gu et al. demonstrated that GLUT4 translocation to the cell membrane was abnormal in CFTR knockout mice muscle fibers (51). Modulating CFTR by ELX/TEZ/IVA could positively influence GLUT4 membrane transportation and improve insulin resistance.

As we found short-term beneficial effects of ELX/TEZ/IVA on glucose tolerance, the “historical” pathophysiology of CFRD caused by β-cell loss cannot be the whole truth. Our data indicate that CFTR modulators might prevent CFRD in CF people—an early use assumed. In our study, participants with IGT and INDET improved; both categories are predictors for CFRD (32). Two subjects with unknown CFRD resolved after initiation of therapy.

In the clinical setting, it might thus be recommended to re-evaluate pathological glucose tolerance after beginning therapy with ELX/TEZ/IVA and before insulin treatment by repeating pathologic OGTTs and repetitive pre- and postprandial glucose measurements.

### Strengths and Limitations

Our study assessed the short-term effect of ELX/TEZ/IVA combination therapy on glucose tolerance in CFTR naïve adolescents with CF. A vast strength is the comprehensive assessment of various outcomes of glucose homeostasis (OGTT, CGM) and other functional and laboratory outcomes. A limitation is the small number of participants included in the study. Therefore, more discrete effects in glucose homeostasis and lung function might not be detected. However, we met the beforehand defined sample size calculation based on our power analysis. It is known that improvement in respiratory function and consecutively in exercise capacity can affect glucose tolerance (52, 53). Our study did not include a detailed physical activity protocol, so we cannot exclude a certain influence of (more) physical activity under ELX/TEZ/IVA treatment on our results.

CGM measurements were performed early after initiation of ELX/TEZ/IVA therapy; thus, we do not know if changes occurred at the time of OGTT measurement. However, as CGM measurements before starting ELX/TEZ/IVA were also normal, we believe a further improvement is unlikely. As we only assessed the short-term effect, we cannot comment on whether the positive effect of ELX/TEZ/IVA on glucose tolerance is transient, sustainable, or if there is even a further improvement. In addition, due to the time range of OGTT (4–10 weeks) after therapy initiation, we cannot explicitly differentiate between very early and later effects. A long-term follow-up of our participants will answer those additional questions.

## Conclusion

This short-term observational study showed that glucose tolerance improves after initiating ELX/TEZ/IVA therapy in teenagers with CF, suggesting that ELX/TEZ/IVA also benefits pancreatic endocrine function. Early treatment with ELX/TEZ/IVA might postpone change future CFRD in adolescents with CF, highlighting that pathological OGTT results should be repeated after initiation or under ELX/TEZ/IVA treatment. While this has to be confirmed in larger longitudinal studies, it supports the early initiation of the CFTR modulator therapy before measurable organ dysfunction occurs, not considering respiratory outcomes alone.

## Data Availability Statement

The raw data supporting the conclusions of this article will be made available by the authors, without undue reservation.

## Ethics Statement

This study was performed in the University Children’s Hospital of Bern, University of Bern, Switzerland, and approved by the Ethics committee of Bern, Switzerland (ID 2021-00982). Written informed consent to participate in this study was provided by the participants’ legal guardian/next of kin.

## Author Contributions

IK: conceptualization, methodology, visualization, writing—original draft, formal analysis, investigation, and data curation. EK: investigation, visualization, and writing—original draft. MB and LK: investigation, writing—review, and editing. CF: methodology, writing—review, and editing. PL: conceptualization, methodology, resources, project administration, writing—review, and editing. CC: conceptualization, writing—review, and editing. CB: conceptualization, methodology, resources, project administration, writing—original draft. All authors contributed to the article and approved the submitted version.

## Conflict of Interest

EK reports personal fees from Sanofi-Aventis. PL reports personal fees from Gilead, Novartis, OM Pharma, Polyphor, Roche, Santhera, Schwabe, Vertex, Vifor, and Zambon and grants from Vertex, all outside the submitted work. The remaining authors declare that the research was conducted in the absence of any commercial or financial relationships that could be construed as a potential conflict of interest.

## Publisher’s Note

All claims expressed in this article are solely those of the authors and do not necessarily represent those of their affiliated organizations, or those of the publisher, the editors and the reviewers. Any product that may be evaluated in this article, or claim that may be made by its manufacturer, is not guaranteed or endorsed by the publisher.
